# Evidence for long-term CD8^+^ memory and functionally diverse CD4^+^ helper responses following yellow fever vaccination: a systematic review

**DOI:** 10.3389/fimmu.2026.1985584

**Published:** 2026-10-14

**Authors:** Ousman L. Camara, Julia Strandmark, Martin R. Goodier, Ed Clarke

**Affiliations:** 1 Vaccines & Immunity Theme, Medical Research Council (MRC) Unit The Gambia at London School of Hygiene & Tropical Medicine (LSHTM), Fajara, Gambia; 2 Clinical Research Unit, Department of Infectious and Tropical Diseases, London School of Hygiene and Tropical Medicine, London, United Kingdom

**Keywords:** 17D, immunity, T cells, vaccination, yellow fever

## Abstract

Yellow fever (YF) 17D-lineage vaccines are among the most effective vaccines ever developed, providing long-term protection traditionally attributed to neutralizing antibodies. However, growing evidence suggests that cellular immunity also contributes to durable vaccine-induced protection. This systematic review synthesized current evidence on the magnitude, kinetics, durability, and functional characteristics of antigen-specific T-cell responses following YF vaccination. A PRISMA 2020-compliant review, prospectively registered in PROSPERO, was conducted using searches of Ovid MEDLINE, Embase, Global Health, Scopus, Web of Science Collection, ClinicalTrials.gov and WHO ICTRP to identify human studies published between 2000 and 2025. Thirty-Six studies met the inclusion criteria and were synthesized narratively because of substantial methodological heterogeneity. The available evidence indicates that primary YF vaccination induces rapid expansion of activated CD8^+^ T cells that subsequently differentiate into long-lived memory populations detectable for years to decades after vaccination. CD4^+^ T-cell responses were broad and functionally diverse, targeting both structural and non-structural viral proteins and supporting adaptive immunity through helper functions. Circulating T follicular helper-cell responses emerged as important immunological correlates of neutralizing antibody development, while inter-individual variation in cellular immunity appeared to be influenced by factors such as early vaccine viral replication and baseline immune activation. Studies of booster vaccination generally demonstrated weaker cellular responses than those observed after primary vaccination. Overall, the evidence suggests that YF vaccination induces durable cellular immunity alongside established humoral responses, supporting a broader understanding of vaccine-induced protection. Nevertheless, definitive evidence linking specific T-cell responses to long-term clinical protection remains limited, and interpretation is constrained by methodological heterogeneity, predominantly observational study designs, and reliance on peripheral blood-based measurements. Further longitudinal and mechanistic studies are needed to define the cellular correlates of durable protection and clarify the contribution of T-cell immunity to long-term vaccine effectiveness.

**Systematic Review Registration:**

https://www.crd.york.ac.uk/PROSPERO/view/CRD420261430765, identifier CRD420261430765.

## 1 Introduction

Yellow fever remains a clinically significant mosquito borne viral disease, particularly in endemic regions of sub–Saharan Africa and South America, where periodic outbreaks continue to impose a substantial public health burden (1). The live-attenuated yellow fever 17D-lineage vaccines (including 17D, 17DD, and 17D-204) are among the most effective vaccines ever developed, capable of inducing long-lasting protective immunity after a single dose in most individuals (2). Evidence from immunological and epidemiological studies shows that this protection is associated with both humoral and cellular immune responses that may persist for years to decade (3). Despite this success, the precise immunological mechanisms underpinning long term protection remain incompletely understood.

A critical gap in the literature lies in the integration of cellular and humoral immune responses into a coherent mechanistic framework. While antibodies are central to protection, T cell–mediated immunity plays a key role in supporting B cell responses and shaping long term immunity. Circulating T follicular helper (cTfh) cells have emerged as important mediators of this interaction, with their expansion correlating with antibody production following vaccination (4). These findings highlight the importance of CD4^+^ T cell subsets in linking cellular and humoral immunity, although the mechanisms governing long term antibody maintenance remain incompletely understood.

Despite evidence that a single dose of yellow fever vaccine provides long-term protection in most individuals, uncertainty remains regarding the durability of immunity across populations and the potential need for booster vaccination. While protection may persist for decades in many adults, reduced seroprotection has been reported in children, immunocompromised individuals, and some endemic populations (3). These observations have contributed to ongoing debate regarding the necessity and timing of booster vaccination.

Given the growing but highly heterogeneous literature on cellular immunity following yellow fever vaccination, this review was designed to address four primary research questions:

1. What are the magnitude, kinetics, durability, and major functional characteristics of yellow fever vaccine-induced CD4^+^ and CD8^+^ T-cell responses?
2. Which yellow fever virus antigens and epitopes dominate vaccine-induced T-cell responses?
3. How are vaccine-induced T-cell responses associated with humoral immunity and neutralizing antibody production?
4. What methodological limitations and key knowledge gaps exist within the current evidence base?

To address these questions, we systematically synthesized and critically evaluated evidence from human studies investigating antigen-specific T-cell responses following yellow fever vaccination.

## 2 Methods

### 2.1 Reporting framework, review conduct, and documentation context

This systematic review was prepared in accordance with the PRISMA 2020 reporting framework, which emphasizes transparent documentation of the rationale for the review, the methods employed, and the findings obtained, including explicit reporting of the study identification and selection process. The review protocol was prospectively registered with the International Prospective Register of Systematic Reviews (PROSPERO 2026 CRD420261430765) to enhance methodological transparency and reduce the risk of reporting bias (**Appendix 1**). A pilot search was conducted in late 2025 to inform protocol development; formal study selection, data extraction, and evidence synthesis commenced only after PROSPERO registration in 2026.

### 2.2 Eligibility criteria and inclusion logic (population, vaccine, antigen specificity, assays, and reporting)

Eligibility criteria were defined to capture human immunogenicity data following receipt of a licensed yellow fever 17D-ineage vaccine, a term used throughout this review to collectively refer to the 17D, 17DD, and 17D-204 vaccine sub-strains. The primary outcome domain was yellow fever virus specific T cell responses measured at any post-vaccination time point; longitudinal sampling was not required, but quantitative T cell immunogenicity data had to be reported.

To ensure true antigen specificity, studies were required to use either MHC tetramers/multimers or ex vivo stimulation with yellow fever vaccine virus or defined yellow fever antigens (including overlapping peptide pools or peptides), rather than non-specific activation alone. Additionally, included studies had to use assays offering single-cell or near-single-cell resolution suitable for T cell quantification and functional profiling namely tetramer staining, intracellular cytokine staining (ICS), activation-induced marker (AIM) assays, or ELISpot with ELISpot eligible only when using defined antigen stimulation and quantitative outcomes with background handling (e.g., background subtraction).

Only primary research articles were eligible; reviews, commentaries, and conference abstracts lacking full datasets were excluded, and studies involving co-administration of vaccines or interventions were included only when yellow fever–specific outcomes could be disentangled and analyzed independently.

### 2.3 Information sources, database coverage, and reproducible search strategy

Evidence identification was conducted across multiple bibliographic sources to maximize coverage of vaccine immunology and cellular immunophenotyping studies. Literature searches were conducted in Ovid MEDLINE, Embase, Global Health, Scopus, the Web of Science Core Collection, ClinicalTrials.gov and WHO ICTRP.

The search logic combined a yellow fever vaccine concept (including text words for “yellow fever vaccine” and vaccine lineage terms such as “YF-17D,” “17D,” “17DD,” and “17D-204,” as well as product identifiers such as “Stamaril,” “YF-VAX,” and “Arilvax”) with a comprehensive cellular immunology concept that captured T cell populations and functions (including CD4 and CD8 subsets, memory and regulatory phenotypes, activation/proliferation descriptors, cytokine terminology, and assay modalities such as ICS, ELISpot, and tetramer/multimer detection).

Searches were restricted to human records using database human limits and broad human text-word capture, and animal-only evidence was excluded by subtracting animal indexing/terms to reduce non-human retrieval. To maintain focus on primary immunogenicity evidence, records indexed as systematic reviews, meta-analyses, or reviews were excluded. The year 2000 was selected *a priori* because the review sought to synthesize evidence generated using contemporary immunological approaches, including multiparameter flow cytometry, tetramer-based antigen-specific T-cell detection, ELISpot assays, and later systems immunology methods, which became more widely applied in yellow fever vaccine research from this period onward. Earlier studies predominantly focused on serological outcomes and provided limited characterization of cellular immune responses. The final retrieval set was limited to English-language publications from 2000–2025, Resource limitations precluded translation of non-English records (**Appendix 2**).

### 2.4 Study selection process and PRISMA-consistent flow from identification to inclusion

Following export and combination of 866 records across databases and clinical trial registries, duplicates were removed prior to screening, resulting in 397 duplicate records being eliminated. The remaining 469 records proceeded with title and abstract screening, and 400 records were excluded due to not meeting the eligibility criteria. Full texts were sought for 69 reports, which were retrieved and assessed for eligibility and 36 studies were included in the final synthesis. Title and abstract screening were conducted independently by two reviewers. Disagreements were resolved through discussion, and where consensus could not be reached, a third reviewer was consulted for adjudication. The same independent dual-review process was applied during full-text screening until final agreement on included studies was achieved.

### 2.5 Data extraction framework, variables captured, and immunologic prioritization

Data extraction was performed using a structured evidence extraction sheet (**Appendix 3**), which captured study identifiers and core design descriptors (authors, year, setting, vaccine type, population characteristics, sample size, and sampling timepoints), as well as parameters including antigenic stimuli, assay platforms, cellular subsets measured, phenotypic marker panels, cytokine outputs, quantitative results, and study-reported strengths, and limitations.

Extraction was structured to preserve assay-specific meaning rather than force premature harmonization; accordingly, T cell outcomes were retained in the form each study reported (e.g., tetramer frequencies, ICS cytokine-positive fractions, ELISpot SFU, AIM activation frequencies, or high-dimensional cytometry-derived subset shifts), along with reported correlational measures linking cellular readouts to neutralizing antibody titers where available.

The feasibility of conducting a meta-analysis was formally assessed following data extraction and evaluation of outcome comparability across studies. Although quantitative outcomes were reported across many studies, meta-analysis was considered inappropriate because heterogeneity extended beyond statistical variation to major differences in assay platforms, antigenic stimuli, outcome definitions, cellular populations evaluated, and sampling timepoints. Studies reported fundamentally different immunological measures, including tetramer frequencies, intracellular cytokine staining responses, ELISpot spot-forming units, activation-induced marker frequencies, and high-dimensional cellular phenotypes, often using distinct antigen targets and reporting frameworks. Consequently, pooled estimates were judged unlikely to provide biologically meaningful summary measures or valid quantitative comparisons. A structured narrative synthesis was therefore undertaken to summaries and interpret the evidence.

Because the central scientific objective was the synthesis of adaptive cellular immunity following yellow fever vaccination, extracted variables were subsequently organized around CD8^+^ effector and memory kinetics, CD4^+^ helper and Tfh-like responses, and the coupling between T-cell signatures and humoral neutralization outcomes. Quantitative outcomes considered eligible included tetramer or multimer frequencies, intracellular cytokine staining (ICS) responses, ELISpot-derived spot-forming units, activation-induced marker (AIM) frequencies, and correlations between cellular immune parameters and neutralizing antibody titers where reported.

### 2.6 Risks of bias assessment: tool selection, domain logic, and operational consistency

Risk of bias was assessed using design-appropriate critical appraisal tools. Observational and intervention studies were evaluated using National Institutes of Health (NIH) Quality Assessment Tools, while case series and quasi-experimental studies were assessed using Joanna Briggs Institute (JBI) critical appraisal checklists (**Appendix 4**). Assessments focused on participant selection, outcome measurement, completeness of follow-up, control of confounding, statistical analysis, and reporting transparency.

## 3 Results

### 3.1 Study identification, screening, and final inclusion

Following deletion of duplicates, 400 out of 469 records were removed through initial screening of abstracts. Following full text evaluation of remaining records, a final selection of 36 studies met the inclusion criteria and were included in qualitative synthesis (**Figure 1**).

**Figure 1:**
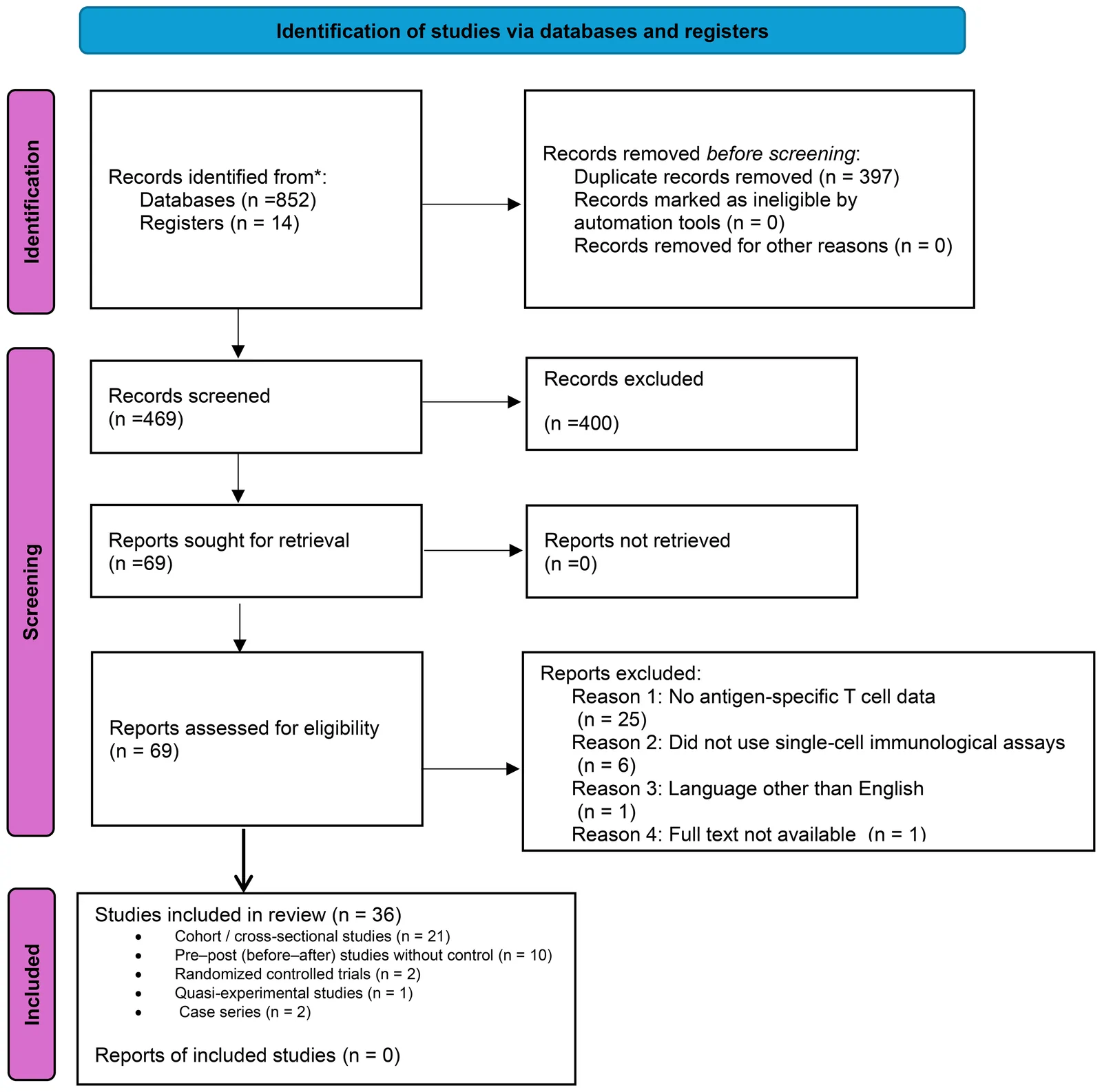
PRISMA 2020 flow diagram of the study selection process. The diagram summarizes the identification, screening, eligibility assessment, and inclusion of studies evaluating antigen-specific T-cell responses following yellow fever 17D-lineage vaccination. A total of 866 records were identified across electronic databases, 397 duplicates were removed, 469 records were screened, 69 full-text articles were assessed for eligibility, and 36 studies were included in the final qualitative synthesis. Data extracted from studies published between 2000 and 2025.

### 3.2 Characteristics of included studies (n = 36)

Across the 36 included studies, the evidence base was dominated by observational immunogenicity cohorts and mechanistic immunology investigations, with only one randomized, double-blind trial using a human challenge framework (**Table 1**). Vaccine products and lineages included 17D/17D-204 (e.g., YF-VAX, Stamaril) and 17DD formulations; studies sampled diverse contexts spanning healthy adults, endemic-area adult populations, pediatric cohorts, immunocompromised patients, and geographically contrasted populations with differing baseline immune activation profiles (**Table 2**).

**Table 1:** Characteristics of the 36 studies included in the review.

Authors	Population	Sample size (n)	Study design	Vaccine type
Fuertes Marraco et al., 2022 (13)	Healthy adults	16	Cross-sectional and Longitudinal cohort	17D
Gaucher et al., 2008 (22)	Healthy adults	40	Longitudinal cohort	17D-204
Miller et al., 2008 (5)	Healthy adults (HLA-A2)	10	Longitudinal cohort	17D
Santos et al., 2008 (24)	First-time vaccinees	12	Longitudinal cohort	17DD
Co et al., 2002 (17)	Healthy adults	4	Case series study	17D
Wieten et al., 2016	Immunocompromised + healthy	45	Retrospective cross-sectional observational	17D
Akondy et al., 2015 (7)	Healthy adults	80	Longitudinal cohort	17D
Mold et al., 2021 (14)	Healthy adults	9	Mechanistic longitudinal	17D
Blom et al., 2013 (9)	Healthy adults	21	Longitudinal cohort	17D
Akondy et al., 2009 (6)	Healthy adults	21	Longitudinal cohort	17D
Koblischke et al., 2017 (26)	Healthy adults	76	Cross-sectional cohort	17D
Co et al., 2009 (10)	HLA-B35 individuals	3	Single-arm longitudinal study	17D
Bovay et al., 2020	Healthy adults	10	Longitudinal cohort	17D
Campi Azevedo et al., 2019	Endemic adults	326	Longitudinal cohort	17DD
Hou et al., 2017 (27)	Healthy adults	21	Longitudinal cohort	17D
Huber et al., 2020 (29)	Healthy adults	34	Longitudinal cohort	17D
Muyanja et al., 2014 (15)	African vs European cohorts	100	Comparative longitudinal cohort	17D
Querec et al., 2009	Healthy adults	25	Longitudinal cohort	17D
Reis LR et al., 2024 (39)	Healthy adults	15	Longitudinal cohort	17DD
Bovay A et al., 2020	Healthy adults (booster)	16	Longitudinal cohort	17D
Wullimann et al., 2025 (28)	Healthy adults	145	Non-randomized clinical trial	17D ± co-admin
Stryhn et al., 2020 (18)	Healthy adults	210	Longitudinal cohort	17D
Wieten et al., 2016	Healthy adults	~100	Cross-sectional cohort	17D
James et al., 2013 (25)	Healthy adults	42	Cross-sectional + Longitudinal cohort	17D
Kalimuddin et al., 2025 (34)	Healthy adults	33	Randomized controlled trial	17D
de Melo et al., 2013 (19)	Healthy adults	220	Cross-sectional	17DD
DeGottardi et al., 2020 (30)	Healthy adults	19	Longitudinal + Cross-sectional cohort	17D
Kongsgaard et al., 2017 (20)	Healthy adults	240	Longitudinal cohort	17D
Campi Azevedo et al., 2019	Children	673	Cross-sectional cohort	17DD
Silva et al., 2011 (37)	Healthy adults	10	Prospective longitudinal observational study	17DD
Akondy et al., 2017 (8)	Healthy adults	37	longitudinal Mechanistic	17D
Konrad et al., 2023 (35)	Healthy adults	51	Cross-sectional observational study	17D
Campi Azevedo et al., 2016 (33)	Endemic adults	171	Cross-sectional (time-stratified)	17DD
Kohler et al., 2012 (23)	Healthy adults	18	Prospective longitudinal observational study	17D
Costa Pereira et al., 2018 (38)	Endemic adults	139	longitudinal dominant + cross sectional	17DD
Slifka et al., 2014 (36)	Atopic dermatitis and non-atopic healthy	82	Randomized controlled trial	17D

**Table 2:** Population and setting characteristics of included studies (n = 36).

Population description	Studies (n)
Population category	
Adults	35
Children/adolescents	1
Adult population subgroups	
Healthy adults	33
Immunocompromised adults	1
Adults with atopic dermatitis	1
Setting	
Endemic settings	7
Non-endemic settings	29

Assay platforms were heterogeneous but consistently met the review’s antigen-specific single-cell-resolution requirements: multiple studies used MHC class I tetramers to quantify CD8^+^ responses, while CD4^+^ antigen specificity was probed using MHC class II tetramers in select cohorts and through epitope mapping/tetramer enrichment approaches (**Table 3**). Functional profiling frequently relied on intracellular cytokine staining (ICS) and/or ELISpot for cytokine-secreting cells, with AIM assays used in recent clinical trial cohort vaccination settings and in the challenge linked randomized trial framework. Time windows sampled ranged from hours and days after vaccination through the acute effector peak around 10–30 days, extending into long-term memory lasting years to decades.

**Table 3:** Immunological assays, T-cell populations assessed, and sampling timepoints across the included studies.

Authors	Assays used	T cell type assessed	Timepoints
Fuertes Marraco et al., 2022 (13)	Flow cytometry staining; scATACseq	CD8^+^ T cells	baseline, 14 days, 6 months, 1–8.6 years
Gaucher et al., 2008 (22)	Flow cytometry; qPCR; microarray; CFSE; multiplex cytokines	CD4^+^ and CD8^+^ T cells	Day 0, 3, 7, 10, 14, 28, 60, 90, 180, and 365
Miller et al., 2008 (5)	Flow cytometry; tetramer; ICS; CFSE; PCR	CD4^+^ and CD8^+^ T cells	Day 0, 3, 7, 11, 15, and 30
Santos et al., 2008 (24)	ELISpot	PBMC-derived T cells	Day 0, 7, 15 and 30
Co et al., 2002 (17)	ELISpot; cytotoxicity assay; proliferation assay	CD8^+^ T cells	Day 0–19 months
Wieten et al., 2016	Tetramer staining; ICS; proliferation assay; PRNT	CD8^+^ T cells	84 days to 18 years
Akondy et al., 2015 (7)	qPCR; flow cytometry; tetramer; microarray	CD8^+^ T cells	Day 0–90
Mold et al., 2021 (14)	scRNA-seq; TCR sequencing; flow cytometry	CD8^+^ T cells	Day 10–1401
Blom et al., 2013 (9)	Flow cytometry; tetramer; ICS; CFSE	CD4^+^ and CD8^+^ T cells	baseline, Day 10, 15, 90
Akondy et al., 2009 (6)	Tetramer; ICS; flow cytometry	CD8^+^ T cells	Day 0–2 years
Koblischke et al., 2017 (26)	ELISpot; ICS; flow cytometry	CD4^+^ T cells	14–54 days
Co et al., 2009 (10)	Tetramer; ICS; flow cytometry	CD8^+^ T cells	Day 0–54 months
Bovay et al., 2020	Flow cytometry; tetramer; PRNT	CD4^+^ and CD8^+^ T cells	Day 0–6 months
Campi Azevedo et al., 2019	Flow cytometry; PRNT; lymphoproliferation	CD4^+^ and CD8^+^ T cells	30–45 days to >10 years
Hou et al., 2017 (27)	Microarray; flow cytometry; PRNT	CD4^+^ and CD8^+^ T cells	4h–168 days
Huber et al., 2020 (29)	Flow cytometry; MHC class II tetramer; FRNT	CD4^+^ T cells	Day 0–28
Muyanja et al., 2014 (15)	RT-PCR; flow cytometry; tetramer; PRNT	CD4^+^ and CD8^+^ T cells	Day 0–1 year
Querec et al., 2009	Microarray; RT-PCR; flow cytometry; PRNT	CD8^+^ T cells	Day 0–90
Reis LR et al., 2024 (39)	CyTOF; Luminex; μFRN	CD4^+^ and CD8^+^ T cells	Day 0–45
Bovay A et al., 2020	PCR; PRNT; flow cytometry; tetramer	CD4^+^ and CD8^+^ T cells	Day 0–6 months
Wullimann et al., 2025 (28)	AIM assay; flow cytometry	CD4^+^ and CD8^+^ T cells	Day 0–30
Stryhn et al., 2020 (18)	ELISpot; ICS; tetramer	CD4^+^ and CD8^+^ T cells	Day 9–41
Wieten et al., 2016	Tetramer; ICS; PRNT	CD8^+^ T cells	Long-term
James et al., 2013 (25)	MHC-II tetramer; ELISpot; proliferation assay	CD4^+^ T cells	Day 0–5 years
Kalimuddin et al., 2025 (34)	ELISpot; AIM; RT-qPCR	CD4^+^ and CD8^+^ T cells	Day 0–56
de Melo et al., 2013 (19)	ELISpot; ICS; flow cytometry	CD4^+^ and CD8^+^ T cells	2 months–4 years
DeGottardi et al., 2020 (30)	CyTOF; tetramer; flow cytometry	CD4^+^ T cells	Day 0–360
Kongsgaard et al., 2017 (20)	Tetramer; ELISpot; ICS; PRNT	CD4^+^ and CD8^+^ T cells	Day 0–2 years
Campi Azevedo et al., 2019	Flow cytometry; PRNT; ICS	CD4^+^ and CD8^+^ T cells	Day 0–10 years
Silva et al., 2011 (37)	ICS; PRNT; flow cytometry	CD4^+^ and CD8^+^ T cells	Day 0–30
Akondy et al., 2017 (8)	Tetramer; sequencing; ATAC-seq	CD8^+^ T cells	Day 0–12 years
Konrad et al., 2023 (35)	Tetramer; cytotoxicity assay; flow cytometry	CD8^+^ T cells	Day 0–100
Campi Azevedo et al., 2016 (33)	Flow cytometry; PRNT; ICS	CD4^+^ and CD8^+^ T cells	0–13 years
Kohler et al., 2012 (23)	Flow cytometry; PRNT; cytokine assay	CD4^+^ and CD8^+^ T cells	Day 0–28
Costa Pereira et al., 2018 (38)	Flow cytometry; PRNT; ICS	CD4^+^ and CD8^+^ T cells	0–11 years
Slifka et al., 2014 (36)	ICS, Flow cytometry, PRNT	CD4^+^ and CD8^+^ T cells	27–43 years

### 3.3 Risk of bias: summary of methodological strengths and vulnerabilities

Risk of bias was assessed using NIH and JBI design-specific tools across 36 included studies, revealing a balanced methodological profile with 18 studies classified as low risk and 18 as moderate risk, and no studies at high risk. (**Appendix 4**). The single randomized controlled trial demonstrated the highest internal validity with a low risk of bias, but its isolated presence limits the overall strength of causal inference within the evidence base.

Across all study designs, a major strength was the consistent use of assays offering single-cell or near-single-cell resolution suitable for immunological analysis (e.g., flow cytometry, tetramer staining, neutralization assays), which enhances confidence in measurement reliability despite other methodological constraints. However, recurrent vulnerabilities included small sample sizes, selective recruitment, and reliance on correlational analyses without comprehensive confounder adjustment, particularly in studies attempting to link immune parameters to clinical outcomes.

### 3.4 CD8^+^ T cell responses after yellow fever vaccination

#### 3.4.1 Dynamics, durability and memory formation

Across multiple cohorts, the primary yellow fever vaccination response consistently shows a rapid expansion of activated and proliferating CD8^+^ T cells, with effector activation signatures peaking in the second to fourth week after vaccination. Early hallmark activation phenotype often summarized by the combined expression of CD38, HLA-DR, Ki-67, and low Bcl-2 captured the effector phase (**Figure 2**) in both classic flow cytometry studies and later mechanistic work (5–8). Quantitatively, dominant epitope-specific responses reach high frequencies in some donors, including tetramer defined responses (6, 9), and functional IFN-γ producing CD8^+^ populations peak robustly during the acute window (5, 6, 10). Many of these measurements were derived from HLA-restricted tetramer systems, particularly HLA-A*02:01- and HLA-B35-restricted epitopes. Importantly, some studies observed that tetramer positivity could precede detectable cytokine function early after vaccination (10). (**Figure 3A**).

**Figure 2:**
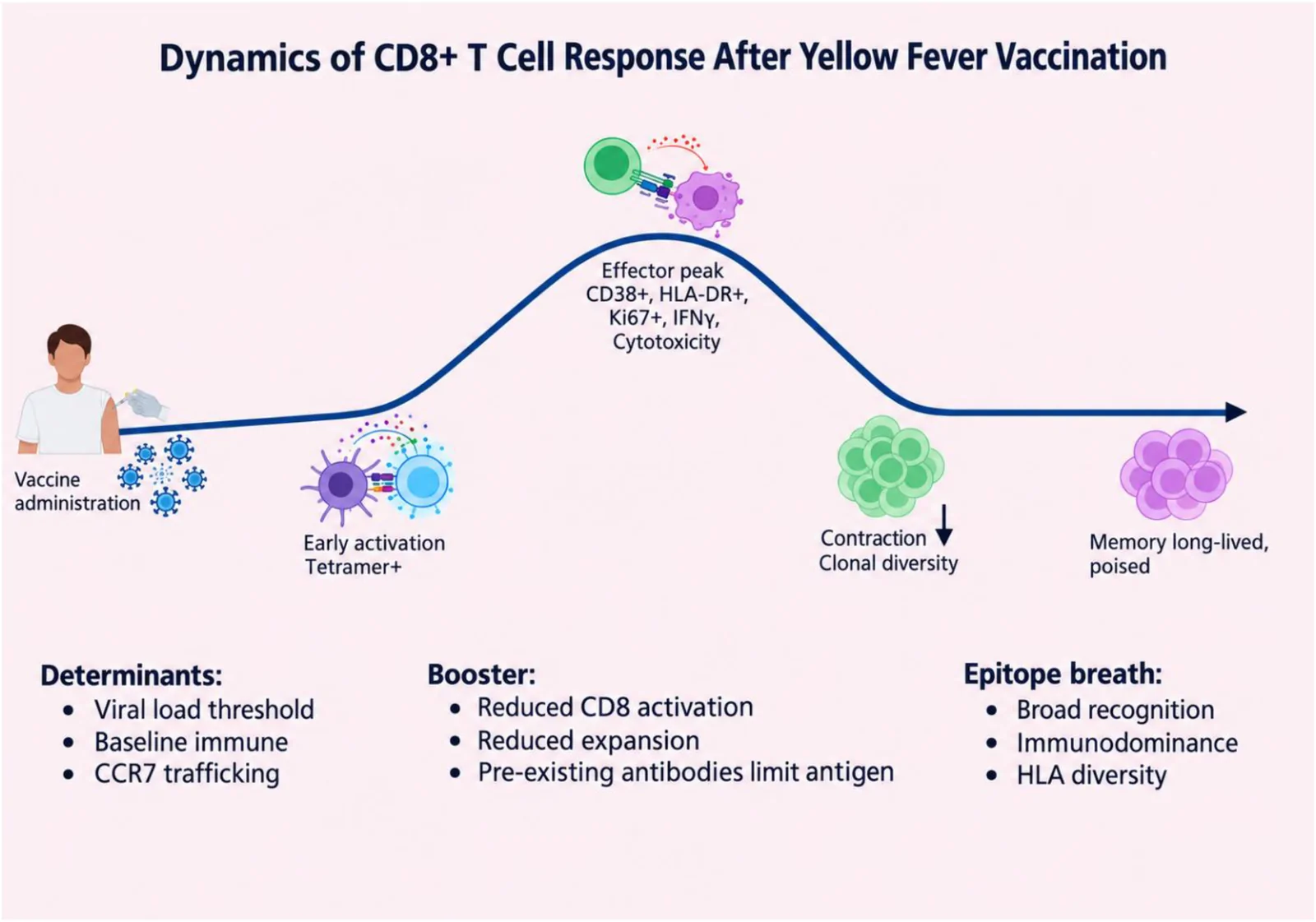
Schematic of CD8^+^ T-cell dynamics after yellow fever vaccination, showing early activation, peak effector expansion, contraction, and persistence of long-lived memory. Response magnitude is influenced by viral load, host factors, booster vaccination, and epitope breadth. Figure created by the authors based on findings reported by Akondy et al. (6), Miller et al. (5), Akondy et al. (7), and Akondy et al. (8). The figure represents a narrative synthesis of published findings and does not reproduce original figures or datasets from the cited studies.

**Figure 3:**
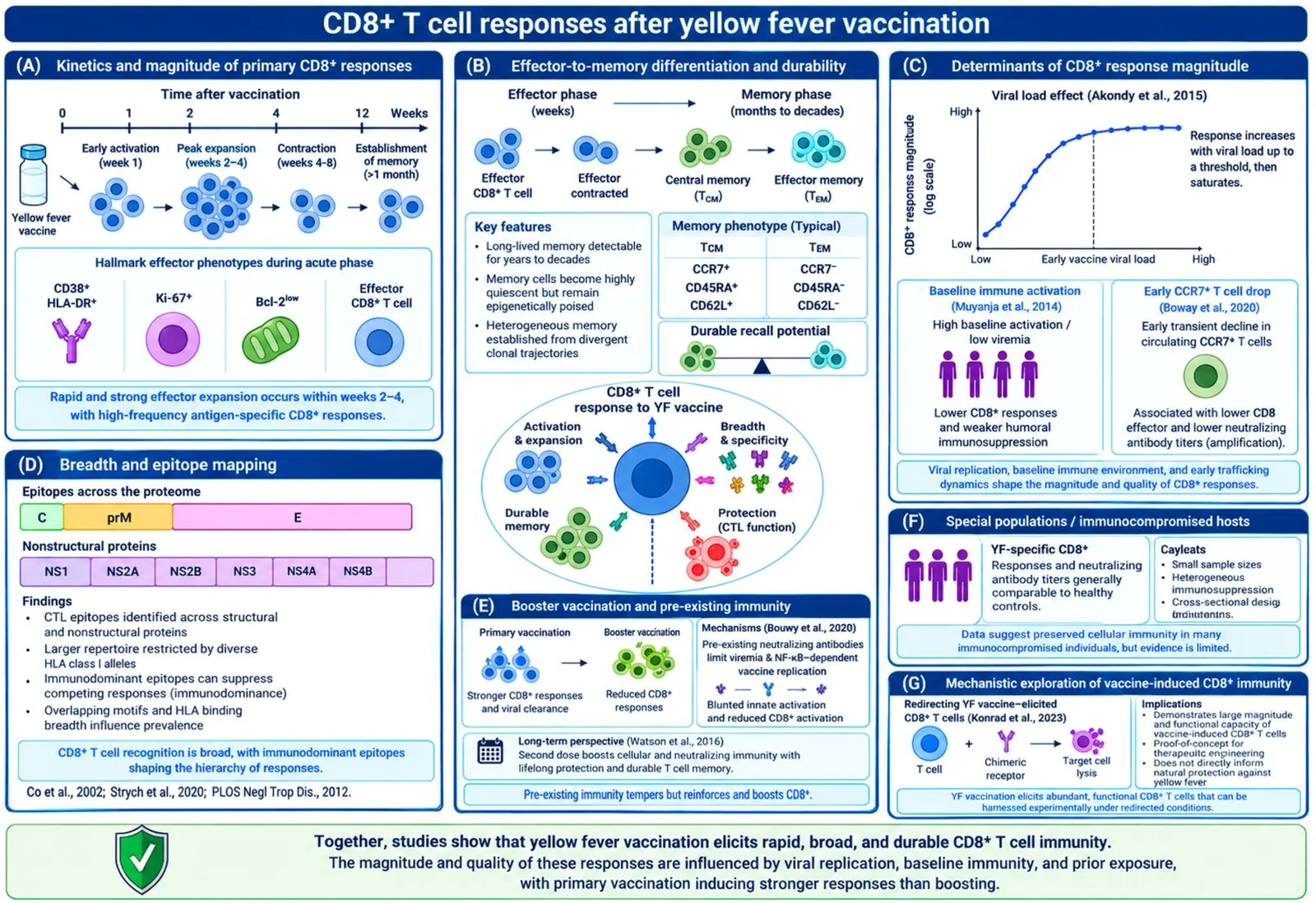
Integrated overview of CD8^+^ T-cell responses following yellow fever vaccination. **(A)** Primary vaccination induces rapid expansion of antigen-specific CD8^+^ T cells, followed by contraction and memory formation. **(B)** Effector cells differentiate into memory populations that can remain detectable over extended periods. **(C)** Response magnitude is influenced by viral replication and host immune status. **(D)** CD8^+^ T cells recognize diverse epitopes across yellow fever virus proteins. **(E)** Booster responses are generally reduced due to pre-existing immunity. **(F)** Immunocompromised individuals can develop YF-specific CD8^+^ T-cell responses, although evidence is limited. Evidence for this observation is derived primarily from a single study. **(G)** Potential implications of vaccine-induced CD8^+^ T-cell responses for understanding immune memory and future vaccine research. Figure created by the authors based on findings reported by Co et al. (17), Miller et al. (5), Akondy et al. (6), Muyanja et al. (15), Akondy et al. (7), Wieten et al. (2016), Akondy et al. (8), Kongsgaard et al. (20), Campi-Azevedo et al. (2019), Stryhn et al. (18), Bovay et al. (2020), Mold et al. (14), Konrad et al. (35), and de Melo et al. (19). The figure represents a narrative synthesis of published findings and does not reproduce original figures or datasets from the cited studies.

A central convergent theme is that yellow fever vaccination is associated with CD8^+^ memory that remains detectable for years and, in some cohorts, for decades. Longitudinal tetramer and functional analyses reports persistence of YF-specific CD8^+^ T cells into extended memory phases (6, 8, 10, 11), and long-term studies in healthy individuals and special populations emphasized that memory can be maintained even as circulating frequencies decline (**Figure 3B**) (11, 12). Mechanistic studies suggest that YF-specific memory CD8^+^ T cells enter a resting state while retaining molecular features that enable rapid reactivation upon re-exposure to antigen (8, 13). Complementing this, single-cell and clonal tracking approaches indicates that memory heterogeneity is established through divergent differentiation trajectories among expanding clones, with contraction accompanied by reduced clonal diversity over time (14).

#### 3.4.2 Determinants of response magnitude and heterogeneity

CD8^+^ response magnitude varies across both individuals and populations and appears to be influenced by viral replication, baseline immune status, and environmental context. A large cohort analysis indicates that early vaccine viral load influences the magnitude of the vaccine-induced CD8^+^ T-cell response up to a threshold, beyond which further increases in viral load do not result in larger responses, suggesting that viral replication constrains CD8^+^ T-cell expansion only within a limited range (7) (**Figure 3C**). In geographically contrasted cohorts, lower viremia and heightened baseline immune activation features are associated with reduced vaccine-induced CD8^+^ responses and weaker humoral outcomes. Distinct baseline immune activation profiles were also observed between the geographically distinct study populations prior to vaccination (15). In another cohort, an early transient drop in circulating CCR7^+^ T cells correlates negatively with later neutralizing antibody titers and with measures of CD8 activation, pointing toward early trafficking dynamics as a potential modifier of downstream adaptive responses (16).

#### 3.4.3 Antigen specificity and immunodominance

Multiple studies have examined the relationship between epitope breadth and immunodominance within YF-induced CD8^+^ T-cell responses. Early epitope mapping identified CTL targets across both structural envelope (E) and nonstructural proteins NS1, NS2b, and NS3, and demonstrated persistence of these responses for months to years (17), Subsequent systematic mapping studies expanded the known epitope repertoire across diverse HLA class I backgrounds while also showing that strongly immunodominant epitopes can suppress responses to competing epitopes, a phenomenon termed immunodomination (18) (**Figure 3D**) Complementary analyses in large, vaccinated cohorts further demonstrated that epitope prevalence is influenced by factors such as HLA-binding characteristics and shared sequence motifs, helping explain patterns of immunodominance across populations (19). The evidence base included both population-scale epitope mapping studies and studies focused on individual HLA-restricted epitopes, particularly HLA-A*02:01-restricted NS4B and HLA-B35-restricted epitopes.

#### 3.4.4 Effects of booster vaccination and special populations

Across multiple lines of evidence, booster vaccination generally elicits markedly reduced CD8^+^ activation and antigen-specific expansion compared with primary vaccination. Large cohort work comparing primary versus booster vaccination documented substantially diminished cellular activation and tetramer-defined expansion in booster recipients (20). Detailed immunoprofiling studies in smaller cohorts further supported that pre-existing neutralizing antibodies can suppress vaccine replication and blunt both innate activation and subsequent CD8^+^ activation during boosting (21) (**Figure 3E**). Observational long-term analyses further showed that booster vaccination neither increased the frequency nor altered the phenotype of long-lived YF-specific memory CD8^+^ T cells in many previously vaccinated adults (11).

In a cohort of 15 immunocompromised individuals and 30 healthy controls evaluated up to 22 years after vaccination, protective neutralizing antibodies were detected in all immunocompromised participants and in 96.7% of controls. Geometric mean neutralizing antibody titers were similar between groups (23.9 vs. 21.7 IU/mL; p = 0.77). The frequency and functional characteristics of YFV-specific CD4+ and CD8+ T-cell responses, including cytokine production and polyfunctionality, were comparable between groups. Neutralizing antibody levels and YFV-specific CD8+ T-cell frequencies decreased with increasing time since vaccination, while early differentiated memory T-cell subsets became relatively more prevalent (12) (**Figure 3F**).

### 3.5 CD4^+^ T cell responses after yellow fever vaccination

#### 3.5.1 Dynamics and functional polarization

CD4^+^ T cell responses were consistently detectable after vaccination and frequently demonstrated mixed functional polarization. Systems-level profiling work described early multilineage activation and CD4 proliferation following vaccination and documented antigen-specific CD4 responses characterized by cytokine production patterns spanning Th1, Th2 and Th17-associated outputs in different individuals (22). Early time-course investigations detected YF-specific cytokine-secreting CD4^+^ cells rapidly after vaccination and linked early CD4 cytokine signatures with later neutralizing antibody outcomes (23) (**Figure 4A**). ELISpot-based studies in primary 17DD vaccinees similarly documented induction of both IFN-γ and IL-4 signatures during the acute post-vaccination window, supporting the observation that early helper responses are not uniformly polarized to a single canonical pattern (24).

**Figure 4:**
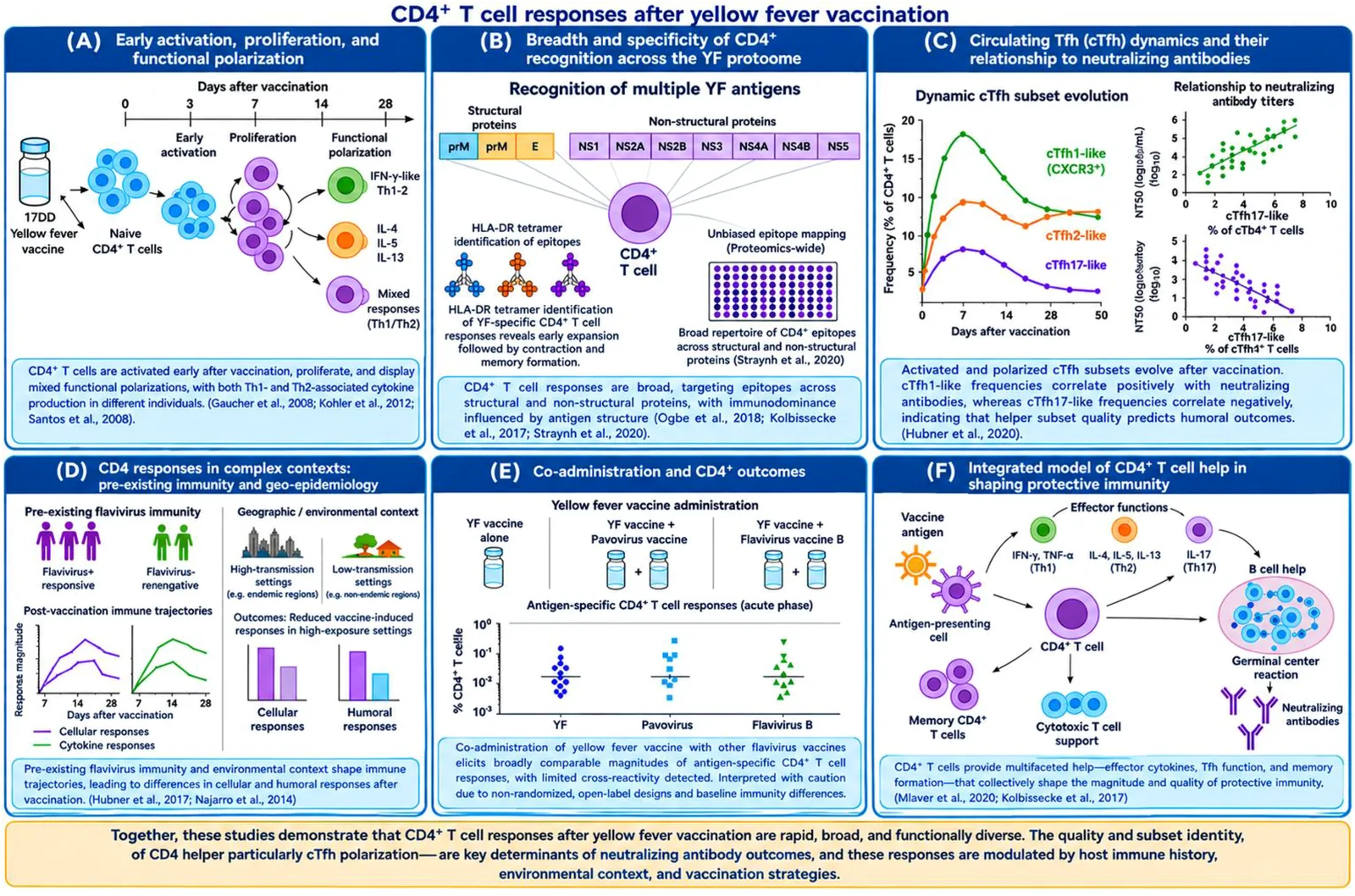
Overview of CD4^+^ T-cell responses following yellow fever vaccination. **(A)** Early activation, proliferation, and functional polarization of CD4^+^ T cells after vaccination. **(B)** Broad CD4^+^ T-cell recognition of epitopes spanning structural and non-structural yellow fever virus proteins. **(C)** Dynamic changes in circulating T follicular helper (cTfh) cell subsets and their association with neutralizing antibody titers. **(D)** Modulation of CD4^+^ T-cell responses by prior flavivirus exposure and environmental context. **(E)** Effects of co-administration with other flavivirus vaccines on antigen-specific T-cell responses. **(F)** Proposed relationship between CD4^+^ T-cell responses and humoral immunity based on the available evidence. Collectively, available evidence suggests that CD4^+^ T-cell responses contribute to the magnitude and quality of immunity induced by yellow fever vaccination. Figure created by the authors based on findings reported by Santos et al. (24), Gaucher et al. (22), Kohler et al. (23), James et al. (25), Muyanja et al. (15), Hou et al. (27), Koblischke et al. (26), Stryhn et al. (18), Huber et al. (29), and Wullimann et al. (28). The figure represents a narrative synthesis of published findings and does not reproduce original figures or datasets from the cited studies.

#### 3.5.2 Antigen recognition and factors influencing CD4^+^ T-cell responses

Multiple studies demonstrated that CD4^+^ T cell recognition after vaccination is broad and spans structural and non-structural proteins. Using HLA-DR tetramers, one study identifies large numbers of naturally processed epitopes and quantified ex vivo dynamics of antigen-specific CD4^+^ T cells peaking early and contracting into memory, with a tendency toward Th1-associated CXCR3 expression among responding cells (25). A complementary cross-sectional mapping study emphasized that the distribution of CD4 immunodominance is shaped by antigen structure, with a pronounced focus on envelope protein regions and differential contributions of structural proteins to the overall CD4 response (26) (**Figure 4B**). At population scale, an unbiased epitope mapping effort also identified substantial repertoires of both CD4^+^ and CD8^+^ epitopes across the proteome, reinforcing the breadth of helper T cell specificity but highlighting practical constraints in fully capturing CD4 repertoires due to lower ex vivo frequencies and HLA-II tetramer availability (18).

In a systems vaccinology cohort, participants with pre-existing seroreactivity to JEV and/or WNV exhibited lower frequencies of myeloid dendritic cells following YF-17D vaccination and reduced TNF-α-secreting T-cell responses, whereas NK-cell activity and IFN-γ production were enhanced (27). A two-cohort comparison demonstrated that baseline immune activation and environmental context can be associated with reduced vaccine-induced cellular and humoral readouts, emphasizing that the “same vaccine” may operate differently across immune landscapes (15) (**Figure 4D**).

A recent open-label, non-randomized trial-cohort evaluated antigen-specific T cell responses when yellow fever vaccine was administered alone versus co-administered with tick-borne encephalitis virus (TBEV) and Japanese encephalitis virus (JEV) vaccines and reported broadly comparable magnitudes of antigen-specific CD4^+^ and CD8^+^ responses across groups, with limited cross-reactivity detected in the tested antigen sets (28) (**Figure 4E**). While reassuring, interpretive confidence is limited by the non-randomized open-label structure and by the likelihood that subtle differences in baseline immunity could still influence group comparisons (28). Consequently, firm conclusions regarding the immunological effects of vaccine co-administration cannot be drawn from this study alone.

### 3.6 Integration of T and B cell immunity: relationships to neutralizing antibodies

A consistent theme across studies that measured both cellular and humoral responses is that helper CD4 biology, particularly Tfh-like features, is more frequently associated with neutralizing antibody outcomes than bulk CD4 or CD8 magnitude alone. A longitudinal profiling focusing on central Tfh (cTfh) cells showed that activated and polarized cTfh subsets evolve after vaccination and that cTfh1-like profiles correlate positively with neutralizing antibody titers (**Figure 4C**). In contrast cTfh17-like frequencies correlate negatively, suggesting that helper subset composition not simply total CD4 activation may be predictive of humoral outcomes (29). High-dimensional cytometry work further supported a dynamic ontogeny of CXCR5^+^ CD4^+^ subsets, with early activated GC-Tfh-like phenotypes transitioning over time, consistent with evolving helper functionality across the acute-to-memory axis (30). Structural immunodominance mapping in the CD4 compartment further reinforced that the envelope protein is a major CD4 target and that Tfh-like cells can provide stronger B cell help than non-Tfh subsets, offering a biologically coherent bridge between antigen structure, helper targeting, and neutralizing antibody outcomes (26) (**Figure 4F**).

Other studies reinforced helper-humoral coupling through early cytokine signatures and longitudinal memory tracking. Early cellular signatures (including cytokine-producing CD4^+^ cells shortly after vaccination) were associated with subsequently high neutralizing antibody titers, consistent with a potential role for early helper programming influencing downstream B cell maturation (23). In endemic-area adult cohorts with revaccination components, both antibody seropositivity and cellular memory features waned over time and were restored by revaccination, suggesting coordinated decay and re-induction of humoral and cellular immunity although the observational design leaves open potential confounding factors such as exposure and undocumented vaccination history (31).

Studies from pediatric endemic populations in region show that both cellular and humoral responses declined markedly in the years following primary vaccination, supporting the argument that durability may be shorter in children than in adults, though the cross-sectional timepoint structure limits causal inference about within-individual decay (32). Long-term adult analyses similarly described waning patterns and complex network reorganizations across years post-vaccination but also raised the possibility of unmeasured exposures or revaccination influencing late timepoint findings (33).

A critical set of correlational studies also linked baseline immune state and early cell trafficking events to antibody outcomes. Heightened baseline immune activation and geographically distinct baseline immune profiles, reflecting differences in environmental exposures, infectious disease burden, and other population-specific factors, are associated with lower vaccine induced neutralizing antibody titers and cellular responses (15), while an early drop in circulating CCR7^+^ T cells correlated with lower neutralizing antibody titers and weaker CD8 activation (16).

### 3.7 T cell–mediated control independent of neutralizing antibodies

A particularly important theme emerging from the available evidence is the potential role of vaccine-induced T-cell responses in viral control when neutralizing antibody responses are limited. In a randomized, double-blind trial employing an Orthoflavivirus challenge framework, stronger vaccine-induced capsid-specific cellular responses were associated with reduced or absent post-challenge viraemia. Moreover, a subset of participants demonstrated viral control despite the absence of detectable seroconversion. These findings suggest that T-cell-mediated mechanisms may contribute to viral control under certain conditions, although direct evidence remains limited to this challenge study and further investigation is required to determine the generalizability of these observations (34).

## 4 Discussion

This systematic review synthesized evidence from 36 studies investigating cellular immune responses following yellow fever (YF) 17D-lineage vaccination. Overall, the evidence demonstrated that YF vaccination induces a coordinated adaptive immune response characterized by robust CD8^+^ and CD4^+^ T-cell activation, the generation of antigen-specific effector populations, and the establishment of long-lived immunological memory. Across studies, a consistent feature of YF vaccination was the generation of large antigen-specific CD8^+^ T-cell responses that contracted into long-lived memory populations, alongside broad and functionally diverse CD4^+^ T-cell responses that remained detectable long after vaccination. These cellular responses occurred alongside the induction of neutralizing antibodies, supporting the concept that YF vaccination generates integrated cellular and humoral immunity.

The reviewed evidence confirms that YF vaccination is a potent inducer of CD8^+^ T-cell immunity. Multiple studies demonstrated substantial expansion of activated and proliferating CD8^+^ T cells during the acute phase following vaccination, with some antigen-specific populations reaching frequencies rarely observed following vaccination and more commonly associated with natural viral infections (5–7, 9, 10, 18, 35). Long-term follow-up studies further showed persistence of YF-specific memory T cells for years to decades after vaccination, while mechanistic investigations suggested that these memory populations retain the capacity for rapid functional recall despite contraction of circulating effector populations over time (8, 10, 11, 14, 22). Collectively, these findings support the durability of vaccine-induced cellular immunity and reinforce the value of YF vaccination as a model for studying long-term human T-cell memory.

Evidence regarding factors influencing the magnitude of cellular responses suggests an important role for both viral replication and host immune context. Studies examining vaccine viremia indicated that early viral replication is associated with subsequent CD8^+^ T-cell expansion, supporting the concept that antigen availability contributes to response magnitude (7). In addition, baseline immune activation and population-specific immune characteristics were associated with variation in both cellular and humoral vaccine responses (15, 16). Geographically distinct baseline immune profiles, characterized by higher frequencies of activated and differentiated immune cell subsets and pro-inflammatory monocytes prior to vaccination, were associated with reduced vaccine-virus replication, lower neutralizing antibody titers, and diminished cellular responses (15), while early changes in circulating CCR7^+^ T-cell populations were linked to subsequent differences in antibody and CD8^+^ T-cell outcomes (16). A randomized study using intracellular cytokine staining after ex vivo stimulation with purified YFV-17D antigen demonstrated measurable YFV-specific CD4^+^ and CD8^+^ T-cell responses following vaccination. While subcutaneous vaccination elicited similar antigen-specific responses in participants with and without atopic dermatitis, transcutaneous vaccination was associated with reduced YFV-specific T-cell responses in individuals with atopic dermatitis (36). Together, these findings suggest that pre-existing immune conditions may contribute to inter-individual variability in vaccine responses.

The review also highlights the importance of CD4^+^ T-cell responses in shaping vaccine-induced immunity. Studies consistently documented induction of antigen-specific CD4^+^ T cells following vaccination and demonstrated considerable diversity in helper-cell phenotypes and cytokine profiles (22, 24–26, 37). Epitope-mapping studies further showed broad CD4^+^ recognition across structural and non-structural viral proteins, with evidence that both antigen structure and HLA diversity influence patterns of immunodominance (18, 25, 26). Importantly, studies investigating circulating T follicular helper (cTfh) cells suggested that the quality and composition of CD4^+^ helper responses may be more strongly associated with neutralizing antibody production than the overall magnitude of CD8^+^ T-cell activation (23, 26, 29, 30). These observations are consistent with an association between helper T-cell function and humoral immune responses following vaccination.

Several included studies examined the relationship between cellular and humoral immunity, although the strength of this relationship varied across cohorts and study designs. While some evidence suggests coordinated maintenance of cellular memory and neutralizing antibodies over time, particularly following revaccination (31, 33, 38), other studies demonstrated persistent neutralizing antibody responses for decades after a single vaccine dose (11). Evidence from endemic settings and pediatric populations additionally suggested greater waning of both cellular and humoral immunity in some groups (31–33). Taken together, these findings indicate that long-term immune durability may differ between populations and that factors such as age, exposure history, and baseline immune status may influence persistence of vaccine-induced protection.

The available evidence also provides insight into booster vaccination and fractional-dose strategies. Studies evaluating booster vaccination showed that secondary vaccination generally induces smaller adaptive responses than primary vaccination, consistent with rapid control of vaccine replication by pre-existing immunity (20, 21). Only one included study directly interrogated fractional dosing (one-fifth dose) using deep cellular phenotyping. That analysis reported robust neutralizing antibody seropositivity and demonstrated measurable expansion and functional modulation of both CD4^+^ and CD8^+^ T-cell compartments following fractional-dose primary vaccination, including shifts toward terminal effector phenotypes and polyfunctional cytokine signatures upon recall stimulation. However, interpretation of equivalence to standard-dose vaccination remains constrained because the fractional-dose cohort lacked a contemporaneous standard-dose comparator and was limited by sample size and follow-up duration, meaning the durability and comparative magnitude of these responses remain uncertain (39). Additional studies in immunocompromised individuals and co-administration settings suggested that vaccine-induced cellular responses can be generated across a range of clinical contexts, although the available evidence remains relatively limited (12, 28).

A particularly important finding emerging from this review is the potential contribution of cellular immunity to viral control independent of neutralizing antibodies. Although most included studies were observational and relied on immunological correlates, the randomized challenge study provided evidence consistent with a contribution of vaccine-induced T-cell responses to control of post-challenge viraemia, including in some individuals lacking detectable challenge-specific neutralizing antibodies. Furthermore, stronger pre-existing T-cell responses were associated with lower viraemia and reduced clinical manifestations following challenge (34). These observations support the view that cellular immunity may represent an important component of protection following YF vaccination and may complement traditional antibody-based measures of vaccine-induced immunity.

The interpretation of these findings should, however, consider several limitations within the available evidence base. Most included studies used observational designs, many involved relatively small cohorts, and immune assessments were largely restricted to peripheral blood samples rather than direct evaluation of tissue-resident or germinal-centre responses. In addition, several mechanistic studies focused on specific HLA-restricted epitopes, which may limit generalizability to broader populations. Nevertheless, the consistency of findings across diverse study designs, populations, and analytical platforms supports the overall conclusion that YF vaccination induces robust and durable cellular immunity that contributes to the generation and maintenance of protective immune responses. Importantly, the heterogeneity observed in this literature was conceptual as well as statistical; many studies measured different immunological constructs using different assay platforms rather than estimating the same outcome under varying conditions. Consequently, direct quantitative pooling would have risked generating summary estimates with limited biological interpretability despite the availability of quantitative data. A further knowledge gap relates to potential differences in immune responses elicited by the major 17D-lineage vaccine sub-strains (17D, 17DD, and 17D-204). Although available evidence suggests possible variation in humoral and cellular immune profiles, the current literature lacks sufficient standardized comparative studies to permit definitive conclusions.

Overall, the evidence indicates that YF 17D-lineage vaccination elicits strong CD8^+^ and CD4 T-cell responses, promotes long-term immunological memory, and generates coordinated cellular and humoral immunity. Future studies incorporating larger and more diverse populations, high-dimensional immune profiling, tissue-based analyses, and standardized immunological endpoints will be important for clarifying mechanisms of protection, understanding variability in long-term immunity, and defining the precise contribution of cellular responses to protection against yellow fever virus infection.

## 5 Limitations

A major limitation of the available evidence is the scarcity of studies directly evaluating protection against yellow fever infection or disease. Most included studies relied on immunological endpoints, including T-cell frequency, phenotype, functional activity, transcriptomic signatures, and neutralizing antibody responses, rather than clinical protection outcomes. Consequently, many proposed correlates of protection and mechanisms of durable immunity remain inferred rather than directly validated in humans.

An important evidence gap identified by this review is the limited availability of data from pediatric and immunocompromised populations. Although a small number of studies evaluated immune responses in children and in individuals with altered immune status, detailed characterization of vaccine-induced cellular immunity remains substantially less developed than in healthy adult populations. Given the importance of these groups for vaccination policy and risk-benefit assessment, further studies are needed to define the magnitude, durability, and functional quality of cellular immune responses following YF vaccination in these populations.

The dominant methodological limitation of this evidence base is the near-total reliance on non-randomized observational designs, often with small sample sizes and immunogenetic restrictions that constrain generalizability. Many “durability” conclusions are drawn from cross-sectional comparisons of different individuals sampled at different times after vaccination, which can confound time-within-individual decay with cohort effects, differential exposure histories, or undocumented boosting (32, 33, 38). Peripheral blood sampling is nearly universal, yet central processes of durable humoral immunity occur in lymph nodes and germinal centers, meaning that much of the “Tfh biology” is inferred from blood proxies rather than measured at the anatomical site of action (26, 29, 30). Another recurrent limitation is the heavy use of single-epitope or single-HLA tracking to represent the CD8 response, 17 studies; this approach provides precision but risks misrepresenting the breadth and population-level heterogeneity of antigen-specific immunity (6–8, 10, 18). Finally, multiple studies infer mechanisms from correlations between baseline immune activation or early trafficking signals and later outcomes, but these associations are highly susceptible to confounding in settings where chronic infections, environmental exposures, and host genetics differ systematically (15, 16, 27).

This review is limited by the heterogeneity of the included literature, which precluded quantitative meta-analysis and necessitated narrative synthesis. Furthermore, the review was restricted to English-language human studies meeting predefined antigen-specific immunological criteria and therefore may not capture all relevant mechanistic evidence from experimental models. Additionally, because the review focused on peer-reviewed literature indexed in major biomedical databases, relevant studies reported only in gray literature sources, or unpublished datasets may not have been captured.

Publication bias cannot be excluded. Formal statistical assessment of publication bias was not undertaken because quantitative meta-analysis was not performed and no common effect measure existed across studies. Nevertheless, studies reporting positive or novel immunological findings may have been more likely to be published than studies reporting null findings, which could influence the available evidence base.

## 6 Future directions

1. Move from correlates to mechanisms using stronger causal designs. The challenge-linked randomized trial (34) demonstrates the kind of design that can anchor mechanistic claims. Similar approaches ethically designed, appropriately powered, and with rigorous immunoprofiling should be prioritized to test whether specific CD4/Tfh or CD8 features causally mediate control.
2. Incorporate tissue-proximal sampling in a realistic way. Fine-needle aspirates of draining lymph nodes or minimally invasive sampling, aligned to acute timepoints, would transform inference about germinal center Tfh and B cell maturation beyond blood proxies (26, 28, 30).
3. Replace single-epitope proxies with multi-epitope, multi-HLA panels. The population-scale mapping study (18) provides a roadmap: future cohorts should use multiplexed tetramer panels (HLA-I and HLA-II) to capture breadth and immunodominance structure rather than extrapolating from one epitope.
4. Standardize outcomes for cross-study comparability. A minimal core outcome set should include: (i) standardized tetramer frequency reporting, (ii) harmonized ICS functional panels (including IL-21 where feasible), (iii) matched PRNT/FRNT neutralization endpoints, and (iv) agreed timepoints (e.g., day 7, 14, 28, 90, 1 year). This would directly address synthesis limitations created by heterogeneous endpoints (5, 6, 11, 20, 22–24, 29).
5. Resolve booster policy contradictions with designs that follow individuals longitudinally. Particularly in children and endemic settings, prospective longitudinal follow-up within the same individuals is essential to disentangle true waning from cohort effects and undocumented boosting (32, 33, 38).
6. Fractional dosing must be tested comparatively and longitudinally. A controlled, parallel standard-dose vs fractional-dose design with multiomics and long-term sampling is needed to determine whether fractional dosing preserves not only early seroconversion but also CD4/Tfh quality, CD8 memory programming, and durability (39).

## 7 Conclusions

The included evidence supports a model of yellow fever vaccine–induced cellular immunity characterized by a strong acute CD8^+^ effector response followed by the persistence of memory populations detectable over extended periods (5, 6, 8, 10, 11), together with broad CD4^+^ helper responses whose quality, particularly Tfh-like polarization, is associated with humoral neutralization outcomes (23, 25, 26, 29, 30). Interpretation of these findings is constrained by the predominance of observational study designs, reliance on peripheral blood measurements, and immunogenetic narrowing (10, 16, 17, 21, 38). The strongest evidence comes from a randomized challenge study, suggesting that T-cell responses may contribute to viral control under specific conditions (34).

## Data Availability

The original contributions presented in the study are included in the article/**Supplementary Material**. Further inquiries can be directed to the corresponding author.

## Supplementary material

Appendix 1 PROSPERO published protocol.

Appendix 2 Database search strategy.

Appendix 3 YF T Cell evidence extraction sheet.

Appendix 4 Study quality assessment.
