# Supplementary material for: Evidence for long-term CD8+ memory and functionally diverse CD4+ helper responses following yellow fever vaccination: a systematic review
Source: Front Immunol. 2026 Oct 14;17:1985584. doi: 10.3389/fimmu.2026.1985584 (PMC13713522; doi:10.3389/fimmu.2026.1985584)
Supplement: Supplementary file 1 [file DataSheet1.pdf]

NIHR | National Institute for Health and Care Research

PROSPERO
International prospective register of systematic reviews

T-cell-mediated immune responses following yellow fever vaccination in humans – Systematic Review

Ousman L Camara, Ed Clarke, Martin Goodier, Julia Strandmark

Citation

Ousman L Camara, Ed Clarke, Martin Goodier, Julia Strandmark. T-cell-mediated immune responses following yellow fever vaccination in humans – Systematic Review . PROSPERO 2026 CRD420261430765. Available from https://www.crd.york.ac.uk/PROSPERO/view/CRD420261430765.

REVIEW TITLE AND BASIC DETAILS

Review title

T-cell-mediated immune responses following yellow fever vaccination in humans – Systematic Review

Condition or domain being studied

Yellow Fever; Yellow Fever Vaccination; Cellular immunity

T Cell Immunity

Rationale for the review

Yellow fever vaccination remains the most effective preventive measure against this life-threatening arboviral disease. While the vaccine is well known for inducing robust neutralizing antibody responses that correlate with protection, accumulating evidence highlights the critical role of T-cell-mediated immunity in shaping the magnitude, durability, and breadth of the protective response.

In particular, T cells contribute to viral clearance, support humoral immunity, and may provide cross-protective mechanisms beyond neutralizing antibodies.

This review aims to synthesize current evidence on T-cell immunity following yellow fever vaccination, focusing on the magnitude, phenotype, and functional characteristics of vaccine-induced responses. Key aspects include the differentiation of effector and memory T-cell subsets, cytokine production profiles, and the development of long-term immunological memory.

Additionally, this review will examine factors influencing T-cell responses, including viral replication dynamics, prior immune status, and vaccine-related variables.

By consolidating available data, this review seeks to provide a comprehensive understanding of the role of T cells in yellow fever vaccine-induced immunity and identify knowledge gaps for

future research.

Review objectives

Primary Objective

To comprehensively synthesize evidence on T-cell-mediated immune responses following yellow fever vaccination, focusing on:

- Magnitude
- Phenotype
- Functional properties
- Role in long-term protection

Primary Research Question

What are the characteristics, dynamics, and functional roles of T-cell responses induced by yellow fever vaccination in humans?

Keywords

Yellow fever; Yellow Fever Vaccine; T cells; Humans

Country

Gambia

ELIGIBILITY CRITERIA

Population

Included
Studies must enroll human subjects who received a licensed Yellow Fever 17D-lineage vaccine (including 17D, 17DD, or 17D-204).

Excluded
Populations that have receive non pre-qualified Yellow fever vaccine or other type of vaccines

Intervention(s) or exposure(s)

Included
Yellow Fever Vaccination

- Yellow fever vaccination (17D lineage)
- Primary, booster, fractional dosing
- Co-administration if YF responses are separable

Excluded

- Experimental/non-17D vaccines
- Passive immunity studies
- In vitro-only studies
- Confounded interventions
- Missing vaccination details

Comparator(s) or control(s)

This review does not have any comparators

Study design

Both randomized and nonrandomized study types will be included.

Included

- Randomized controlled trials
- Non-randomized interventional studies
- Cohort studies
- Cross-sectional studies with quantitative T-cell measurement

Excluded

- Reviews
- Editorials
- Conference abstracts without full data
- Case reports
- Animal/in vitro-only studies

Context

This review will focus on studies conducted in human populations receiving licensed Yellow Fever (YF) vaccines in both clinical and observational settings, including hospital-based studies, vaccination campaigns, and community-based cohorts. Studies from all geographic regions will be considered, with particular relevance to endemic areas in Africa and South America where Yellow Fever vaccination is routinely implemented, as well as non-endemic regions where vaccination is administered to travellers or for outbreak control.

Eligible studies may include participants across diverse immunological backgrounds, including individuals with prior flavivirus exposure, varying age groups, and differing health statuses, provided that YF vaccination is clearly documented and T-cell-mediated immune responses can be specifically attributed to the vaccine.

There will be no restrictions based on healthcare setting, study environment, or vaccination delivery platform, as long as studies report quantitative measurements of antigen-specific T-cell responses following vaccination. Studies limited to laboratory-based in vitro analyses without vaccinated human participants will be excluded

TIMELINE OF THE REVIEW

Date of first submission to PROSPERO

25 June 2026

Review timeline

Review start date: 22 June 2026. Review end date: 22 December 2026.

Date of registration in PROSPERO

26 June 2026

AVAILABILITY OF FULL PROTOCOL

Availability of full protocol

A full protocol has been written and uploaded to PROSPERO. The protocol may be accessed through this link https://www.crd.york.ac.uk/PROSPEROFILES/179394a9fa4d6a070dc34024044827c3.pdf.

SEARCHING AND SCREENING

Search for unpublished studies

Only published studies will be sought.

Main sources that will be searched

The main sources to be searched are *Embase*, *MEDLINE* and *Scopus*.

Other sources that will be searched
Global Health

Web of Science Collection

Search language restrictions

The review will only include studies published in English.

Search date restrictions

Databases will be searched for articles published from 1 January 2000 and before by 1 January 2026.

Other methods of identifying studies

Other studies will be identified by: *contacting authors or experts*, *reference list checking (backward citation searching)* and *searching trial or study registers*.

Link to search strategy

A full search strategy has been uploaded to PROSPERO. The PDF may be accessed through this link https://www.crd.york.ac.uk/PROSPEROFILES/8b356c8805957393eaf242975560937f.pdf.

Selection process

Studies will be screened independently by at least two people (or person/machine combination) with a process to resolve differences.

Other relevant information about searching and screening

Discrepancies resolved by discussion or third reviewer

DATA COLLECTION PROCESS

Data extraction from published articles and reports

Data will be extracted by one person (or a machine) and checked by at least one other person.

Authors will be asked to provide any required data not available in published reports.

Study risk of bias or quality assessment

Risk of bias will be assessed using: *JBI tools*

NIH Quality Assessment tools

Data will be assessed by one person (or a machine) and checked by at least one other person.

Additional information will **not** be sought from study investigators if required information is unclear or unavailable in the study publications/reports.

Reporting bias assessment

Risk of bias due to missing results will not be assessed

Certainty assessment

Certainty of findings will not be assessed

OUTCOMES TO BE ANALYSED

Main outcomes

T cell-mediated immune responses, including but not limited to:

- CD4+ and CD8+ T cell responses: frequency/proportion of YF-specific CD4+ or CD8+ T cells post-vaccine (e.g. by tetramer, ICS, or ELISPOT)
- Memory T cell markers: counts or percentages of memory subsets (e.g. central/effector memory CCR7+/CD45RA-, EMRA) among YF-specific T cells
- Cytokine production: number or proportion of T cells producing cytokines (IFN-y, IL-2, TNF-a, IL-4, IL-5, etc.) upon YF antigen stimulation
- Activation markers: expression of activation (e.g. CD69, HLA-DR) or proliferation markers (e.g. Ki-67) on YF-specific T cells

Additional outcomes

There are no additional outcomes.

PLANNED DATA SYNTHESIS

Strategy for data synthesis

Data from included studies will be combined using a structured narrative synthesis supported by tabulation of key study characteristics and outcomes. Given the anticipated heterogeneity in study design, population characteristics, vaccine formulations, immunological assays, and outcome measures, a formal meta-analysis is not considered appropriate. Instead, findings will be organised thematically to allow comparison across studies while preserving important contextual differences.

Quantitative results relating to T-cell-mediated immune responses will be summarised using tables that present study-level data, including measures of T-cell frequency, phenotype, cytokine production, and activation markers. Where possible, results will be grouped according to common domains such as $CD4^+$ and $CD8^+$ responses, memory T-cell subsets, and functional profiles.

Semi-quantitative comparisons will be performed to identify consistent patterns, trends, and ranges of reported responses across studies. These comparisons may include the direction and magnitude of observed effects, relative differences between populations (e.g. age groups or prior flavivirus exposure), and temporal dynamics of immune responses following vaccination.

Without pooling effect sizes statistically, this approach will allow the identification of convergence or divergence in findings across heterogeneous datasets.

Narrative synthesis will also integrate contextual factors influencing outcomes, such as differences in assay methodologies, sampling time points, and vaccination strategies. This will facilitate interpretation of variability between studies and help identify key determinants of T-cell response heterogeneity.

Overall, this approach is intended to provide a comprehensive and transparent synthesis of available evidence while maintaining methodological rigor in the absence of sufficient homogeneity required for formal quantitative meta-analysis

CURRENT REVIEW STAGE

Stage of the review at this submission

Review stage	Started	Completed
Pilot work	✓	✓
Formal searching/study identification		
Screening search results against inclusion criteria		
Data extraction or receipt of IPD		
Risk of bias/quality assessment		
Data synthesis		

Review status

The review is currently planned or ongoing.

Publication of review results

Results of the review will be published in English.

REVIEW AFFILIATION, FUNDING AND PEER REVIEW

Review team members

Mr Ousman L Camara (review guarantor and contact) ORCID: 0009-0006-4211-1181. Medical Research Council Unit The Gambia at London School of Hygiene and Tropical Medicine. Gambia.

No conflict of interest declared.

Professor Ed Clarke. Medical Research Council Unit The Gambia at London School of Hygiene and Tropical Medicine. Gambia.

No conflict of interest declared.

Assistant Professor Martin Goodier. Medical Research Council Unit The Gambia at London School of Hygiene and Tropical Medicine. Gambia.

No conflict of interest declared.

Assistant Professor Julia Strandmark. Medical Research Council Unit The Gambia at London School of Hygiene and Tropical Medicine. Gambia.. Gambia.

No conflict of interest declared.

Named contact

Mr Ousman L Camara (Ousman-L.CAMARA@lshtm.ac.uk). ORCID: 0009-0006-4211-1181. Medical Research Council Unit The Gambia at London School of Hygiene and Tropical Medicine. Gambia.

Review affiliation

Medical Research Council Unit The Gambia at London School of Hygiene and Tropical Medicine.

Funding source

Review has no funding and no agreed support from an academic institution and is done in authors' own time.

Peer review

There has been no peer review of this planned review.

ADDITIONAL INFORMATION

Additional information

The protocol has not undergone formal external peer review. However, it has been reviewed internally by the review team, including senior researchers with extensive expertise in immunology and vaccine research.

Review conflict of interest

Declared individual interests are recorded under team member details.. No additional interests are recorded for this review.

Medical Subject Headings

Humans; Immunity; T-Lymphocytes; Vaccination; Yellow Fever

SIMILAR REVIEWS

Check for similar records already in PROSPERO

PROSPERO identified a number of existing PROSPERO records that were similar to this one (last check made on 22 June 2026). These are shown below along with the reasons given by that the review team for the reviews being different and/or proceeding.

- Yellow Fever Vaccination in People Living with HIV: An Updated Systematic Review and Meta-analysis of Immunogenicity, Durability of Protection, and Safety [published 11 June 2026] [CRD420261420807]. The review was judged **not to be similar**
- Immunogenicity of yellow fever re-vaccination: a systematic literature review [published 7 May 2024] [CRD42024540804]. The review was judged **not to be similar**
- Long-term immunity of yellow fever vaccination [published 27 March 2023] [CRD42023384087]. The review was judged **not to be similar**

PROSPERO version history

- Version 1.0, published 26 Jun 2026

Disclaimer

The content of this record displays the information provided by the review team. PROSPERO does not peer review registration records or endorse their content.

PROSPERO accepts and posts the information provided in good faith; responsibility for record content rests with the review team. The guarantor for this record has affirmed that the information provided is truthful and that they understand that deliberate provision of inaccurate information may be construed as scientific misconduct.

PROSPERO does not accept any liability for the content provided in this record or for its use. Readers use the information provided in this record at their own risk.

Any enquiries about the record should be referred to the named review contact
