# Supplementary material for: Evidence for long-term CD8+ memory and functionally diverse CD4+ helper responses following yellow fever vaccination: a systematic review
Source: Front Immunol. 2026 Oct 14;17:1985584. doi: 10.3389/fimmu.2026.1985584 (PMC13713522; doi:10.3389/fimmu.2026.1985584)
Supplement: Supplementary file 2 [file DataSheet2.pdf]

Database and Registry search strategies.

Ovid MEDLINE(R) ALL <1946 to January 13, 2026>

Search date: 27 June 2026

1 (yellow fever vaccin* or YF vaccin*).tw. 1258

2 (YF-17D or 17D or 17DD or 17D-204).tw. 1122

3 (Stamaril or YF-VAX or Arilvax).tw. 64

4 (live attenuated yellow fever vaccine or LAYFV).tw. 40

5 exp Yellow Fever Vaccine/ 1032

6 1 or 2 or 3 or 4 or 5 2262

7 exp T-Lymphocytes/ 389054

8 (T cell* or T-cell* or T lymphocyte* or T-lymphocyte*).tw. 554416

9 (CD4* T cell* or CD4* lymphocyte* or T helper cell* or helper T cell*).tw. 97311

10 (CD8* T cell* or CD8* lymphocyte* or cytotoxic T cell* or killer T cell*).tw. 96510

11 (memory T cell* or T memory cell*).tw.12684

12 (regulatory T cell* or Treg* or suppressor T cell*).tw. 56717

13 (Th1 or Th2 or Th17).tw. 80736

14 exp Lymphocyte Activation/ 117553

15 (T cell activation or T cell proliferation or T cell response*).tw. 83214

16 (cytokine* adj3 (T cell* or production or secretion)).tw. 86373

17 (interferon gamma or IFN-g or TNF-alpha or tumor necrosis factor alpha or IL-2 or interleukin-2).tw. 346007

18 (IL-4 or IL-5 or IL-10 or IL-13 or IL-17).tw. 149221

19 (intracellular cytokine staining or ICS or ELISPOT or enzyme-linked immunospot).tw. 21536

20 (tetramer* or MHC multimer* or MHC class I or MHC class II).tw. 58078

21 (cytotoxic* or cytolytic or degranulation or perforin or granzyme).tw. 430339

22 exp Immunity, Cellular/ 179079

23 (cellular immunity or cell-mediated immunity).tw. 27721

24 exp Immunologic Memory/ 20122

25 (immunological memory or immune memory).tw. 5579

26 (adaptive immunity or acquired immunity).tw. 21457

27 (immunogenicity or immune response*).tw. 402494

28 7 or 8 or 9 or 10 or 11 or 12 or 13 or 14 or 15 or 16 or 17 or 18 or 19 or 20 or 21 or 22 or 23 or 24 or 25 or 26 or 27 1682847

29 exp Humans/ 23204388

30 (human or humans or patient* or participant* or volunteer* or subject* or donor*).tw. 14204284

31 29 or 30 26173657

32 6 and 28 and 31 528

33 exp Animals/ 28618824

34 (animal or animals or mouse or mice or murine or rat or rats or rodent*).tw. 4057358

35 33 or 34 29036254

36 32 not 35 29

37 (systematic review or meta-analysis or review).pt. 3821103

38 36 not 37 24

39 limit 38 to english language 24

40 limit 39 to yr="2000 - 2025" **21**

Embase Classic+Embase <1947 to 2026 January 12>

Search date: 27 June 2026

1 exp Yellow Fever Vaccine/ 3566

2 (yellow fever vaccin* or YF vaccin*).tw. 1683

3 (YF-17D or 17D or 17DD or 17D-204).tw. 1660

4 (Stamaril or YF-VAX or Arilvax).tw. 329

5 (live attenuated yellow fever vaccine or LAYFV).tw. 53

6 1 or 2 or 3 or 4 or 5 5009

7 exp T-Lymphocytes/ 790759

8 (T cell* or T-cell* or T lymphocyte* or T-lymphocyte*).tw. 797672

9 (CD4* T cell* or CD4* lymphocyte* or T helper cell* or helper T cell*).tw. 141016

10 (CD8* T cell* or CD8* lymphocyte* or cytotoxic T cell* or killer T cell*).tw. 148244

11 (memory T cell* or T memory cell*).tw.20833

12 (regulatory T cell* or Treg* or suppressor T cell*).tw. 93638

13 (Th1 or Th2 or Th17).tw. 120800

14 exp Lymphocyte Activation/ 38608

15 (T cell activation or T cell proliferation or T cell response*).tw. 122819

16 (cytokine* adj3 (T cell* or production or secretion)).tw. 124483

17 (interferon gamma or IFN-g or TNF-alpha or tumor necrosis factor alpha or IL-2 or interleukin-2).tw. 444653

18 (IL-4 or IL-5 or IL-10 or IL-13 or IL-17).tw. 215073

19 (intracellular cytokine staining or ICS or ELISPOT or enzyme-linked immunospot).tw. 42014

20 (tetramer* or MHC multimer* or MHC class I or MHC class II).tw. 70602

21 (cytotoxic* or cytolytic or degranulation or perforin or granzyme).tw. 567824

22 exp Immunity, Cellular/ 119491

23 (cellular immunity or cell-mediated immunity).tw. 36509

24 exp Immunologic Memory/ 11541

25 (immunological memory or immune memory).tw. 7628

26 (adaptive immunity or acquired immunity).tw. 29116

27 (immunogenicity or immune response*).tw. 546304

28 7 or 8 or 9 or 10 or 11 or 12 or 13 or 14 or 15 or 16 or 17 or 18 or 19 or 20 or 21 or 22 or 23 or 24 or 25 or 26 or 27 2321980

29 exp Humans/ 31012064

30 (human or humans or patient* or participant* or volunteer* or subject* or donor*).tw. 20833918

31 29 or 30 34002639

32 6 and 28 and 31 1097

33 exp Animals/ 37820592

34 (animal or animals or mouse or mice or murine or rat or rats or rodent*).tw. 5337891

35 33 or 34 38160490

36 32 not 35 16

37 (systematic review or meta-analysis or review).pt. 3527485

38 36 not 37 11

39 limit 38 to english language 11

40 limit 39 to yr="2000 - 2025" **11**

Global Health <1910 to 2026 Week 02>

Search date: 27 June 2026

1 (yellow fever vaccin* or YF vaccin*).tw. 1392

2 (YF-17D or 17D or 17DD or 17D-204).tw. 936

3 (Stamaril or YF-VAX or Arilvax).tw. 44

4 (live attenuated yellow fever vaccine or LAYFV).tw. 31

5 1 or 2 or 3 or 4 1897

6 (T cell* or T-cell* or T lymphocyte* or T-lymphocyte*).tw. 89827

7 (CD4* T cell* or CD4* lymphocyte* or T helper cell* or helper T cell*).tw. 47986

8 (CD8* T cell* or CD8* lymphocyte* or cytotoxic T cell* or killer T cell*).tw. 20016

9 (memory T cell* or T memory cell*).tw.1888

10 (regulatory T cell* or Treg* or suppressor T cell*).tw. 7757

11 (Th1 or Th2 or Th17).tw. 21109

12 exp Lymphocyte Activation/ 6946

13 (interferon gamma or IFN-g or TNF-alpha or tumor necrosis factor alpha or IL-2 or interleukin-2).tw. 95783

14 (IL-4 or IL-5 or IL-10 or IL-13 or IL-17).tw. 40423

15 (intracellular cytokine staining or ICS or ELISPOT or enzyme-linked immunospot).tw. 4930

16 (tetramer* or MHC multimer* or MHC class I or MHC class II).tw. 4347

17 (cytotoxic* or cytolytic or degranulation or perforin or granzyme).tw. 128802

18 (cellular immunity or cell-mediated immunity).tw. 18566

19 (immunological memory or immune memory).tw. 1316

20 (adaptive immunity or acquired immunity).tw. 5986

21 (immunogenicity or immune response*).tw. 177518

22 6 or 7 or 8 or 9 or 10 or 11 or 12 or 13 or 14 or 15 or 16 or 17 or 18 or 19 or 20 or 21 442803

23 exp Humans/ 3826877

24 (human or humans or patient* or participant* or volunteer* or subject* or donor*).tw. 4775009

25 23 or 24 5014490

26 5 and 22 and 25 424

27 (animal or animals or mouse or mice or murine or rat or rats or rodent*).tw. 5129227

28 26 not 27 21

29 (systematic review or meta-analysis or review).mp. [mp=abstract, title, original title, heading words, cabicodes words] 542846

30 28 not 29 19

31 limit 30 to english language 18

32 limit 31 to yr="2000 - 2025" **16**

Scopus

Search date: 28 June 2026

TITLE-ABS-KEY (Yellow fever T Cell response) AND (LIMIT-TO (EXACTKEYWORD , "Human") OR LIMIT-TO (EXACTKEYWORD , "Humans") OR LIMIT-TO (EXACTKEYWORD , "Yellow Fever Vaccine") OR LIMIT-TO (EXACTKEYWORD , "Article") OR LIMIT-TO (EXACTKEYWORD , "Cd8+ T Lymphocyte") OR LIMIT-TO (EXACTKEYWORD , "Yellow Fever Virus") OR LIMIT-TO (EXACTKEYWORD , "T Lymphocyte") OR LIMIT-TO (EXACTKEYWORD , "Cellular Immunity") OR LIMIT-TO (EXACTKEYWORD , "Cd4+ T Lymphocyte") OR LIMIT-TO (EXACTKEYWORD , "Adaptive Immunity") OR LIMIT-TO (EXACTKEYWORD , "Vaccine") OR LIMIT-TO (EXACTKEYWORD , "Cd8-positive T-lymphocytes") OR LIMIT-TO (EXACTKEYWORD , "T Lymphocyte Activation") OR LIMIT-TO (EXACTKEYWORD , "T-lymphocytes") OR LIMIT-TO (EXACTKEYWORD , "Cd4-positive T-lymphocytes")) **385**

Web of Science Collection

Search date: 28 June 2026

yellow fever T cell response (Topic) and Yellow Fever Vaccine (OR – Search within topic) and Yellow Fever Virus (OR – Search within topic) and 17dd Vaccine (OR – Search within topic) and Yellow Fever Vaccination (OR – Search within topic) and Review Article (Exclude – Document Types) and Enriched Cited References (Exclude) and Open publisher-invited reviews (Exclude) and 2025 or 2024 or 2023 or 2021 or 2022 or 2020 or 2018 or 2019 or 2016 or 2017 or 2015 or 2014 or 2013 or 2012 or 2011 or 2010 or 2009 or 2008 or 2007 or 2006 or 2005 or 2004 or 2003 or 2002 or 2001 or 2000 (Publication Years) and Meeting Abstract or Editorial Material or Book Chapters or Letter (Exclude – Document Types) and Proceeding Paper or Correction or News Item or Reprint (Exclude – Document Types) and Data Paper or Biographical-Item (Exclude – Document Types) and German or Portuguese or Spanish or French (Exclude – Languages) and Immunology or Infectious Diseases or Tropical Medicine (Research Areas) and All Open Access (Open Access) and Social Sciences Citation Index (SSCI) (Exclude – Web of Science Index) **419**

https://www.webofscience.com/wos/woscc/summary/5bf225c5-3e1d-4c36-8409-6575a014522d-01caab0251/relevance/1

ClinicalTrials.gov

Search date: 25 September 2026

Yellow fever, T cells, Cellular Immunity, Yellow fever vaccine, Yellow fever vaccination	**14**

WHO ICTRP

Search date: 25 September 2026

Yellow fever, T cells, Cellular Immunity, Yellow fever vaccine, Yellow fever vaccination	**0**
