# Supplementary material for: Evidence for long-term CD8+ memory and functionally diverse CD4+ helper responses following yellow fever vaccination: a systematic review
Source: Front Immunol. 2026 Oct 14;17:1985584. doi: 10.3389/fimmu.2026.1985584 (PMC13713522; doi:10.3389/fimmu.2026.1985584)
Supplement: Supplementary file 3 [file DataSheet3.pdf]

NIH Quality Assessment Tool for Before-After (Pre-Post) Studies With No Control Group

No	Authors	Q1	Q2	Q3	Q4	Q5	Q6	Q7	Q8	Q9	Q10	Q11	Q12
1	Fuertes Marraco et al., 2022	+	-	+	+	-	+	+	-	?	+	+	NA
2	Gaucher et al., 2008	+	-	-	-	+	+	+	+	-	+	+	NA
3	Miller et al., 2008	+	+	-	?	-	+	+	?	+	+	+	NA
4	Santos et al., 2008	+	+	-	?	-	+	+	-	?	+	+	NA
5	Akondy et al., 2015	+	+	-	-	+	+	+	?	?	+	+	NA
6	Mold et al., 2021	+	+	-	-	-	+	+	?	-	+	+	NA
7	Blom et al., 2013	+	+	-	-	+	+	+	-	?	+	+	NA
8	Akondy et al., 2009	+	+	-	-	+	+	+	?	+	+	+	NA
9	Bovay et al., 2020	+	+	-	-	-	+	+	?	?	+	+	NA
10	Reis LR et al., 2024	+	+	+	+	-	+	+	+	?	+	+	NA
11	Co et al., 2009	+	-	-	?	-	+	+	?	+	+	+	NA

NIH Quality Assessment Tool for Observational Cohort and Cross-Sectional Studies

No	Authors	Q1	Q2	Q3	Q4	Q5	Q6	Q7	Q8	Q9	Q10	Q11	Q12	Q13	Q14
1	Wieten et al., 2016	+	+	-	+	-	+	+	+	+	+	+	?	?	+
2	Koblischke et al., 2017	+	+	-	+	-	+	+	+	+	-	+	?	+	+
3	Campi-Azevedo et al., 2019	+	+	-	+	+	+	+	+	+	+	+	-	-	+
4	Hou et al., 2017	+	+	-	+	-	+	+	+	+	+	+	?	-	+
5	Huber et al., 2020	+	+	-	+	-	+	+	+	+	+	+	-	?	+
6	Muyanja et al., 2014	+	+	-	+	+	+	+	+	+	+	+	?	-	+
7	Querec et al., 2009	+	+	-	+	-	+	+	-	+	+	+	?	-	+
8	Bovay A et al., 2020	+	+	-	+	-	+	+	+	+	+	+	?	?	+
9	Stryhn et al., 2020	+	+	-	+	-	+	+	-	+	+	+	?	+	?
10	Wieten et al., 2016	+	+	-	+	-	+	+	-	+	-	+	?	?	?
11	James et al., 2013	+	+	+	+	-	+	+	-	+	+	+	-	-	+
12	de Melo et al., 2013	+	+	+	+	-	+	+	-	+	-	+	-	-	?

13	DeGottardi et al., 2020	+	+	+	+	+	+	+	-	+	+	+	-	-	?
14	Kongsgaard et al., 2017	+	+	+	+	-	+	+	+	+	+	+	?	+	?
15	Campi-Azevedo et al., 2019	+	+	+	+	-	+	+	+	+	-	+	+	+	?
16	Silva et al., 2011	+	+	+	+	-	+	+	-	+	+	+	?	?	?
17	Akondy et al., 2017	+	+	-	+	-	+	+	-	+	+	+	-	-	?
18	Konrad et al., 2023	+	+	-	+	-	+	+	-	+	-	+	?	+	?
19	Campi-Azevedo et al., 2016	+	+	+	+	-	+	+	+	+	-	+	-	+	?
20	Kohler et al., 2012	+	+	-	+	-	+	+	-	+	+	+	-	-	?
21	Costa-Pereira et al., 2018	+	+	-	+	-	+	+	+	+	+	+	-	+	?

NIH Quality Assessment of Controlled Intervention Studies

No	Authors	Q1	Q2	Q3	Q4	Q5	Q6	Q7	Q8	Q9	Q10	Q11	Q12	Q13	Q14
1	Kalimuddin et al., 2025	+	+	+	+	+	+	+	+	+	+	+	+	+	+
2	Slifka et al., 2014	+	+	+	+	+	+	+	+	+	+	+	+	+/-	+

JBI Critical Appraisal Checklist for Case Series

No	Authors	Q1	Q2	Q3	Q4	Q5	Q6	Q7	Q8	Q9	Q10
1	Co et al., 2002	-	+	+	-	+	-	-	+	+	+

JBI Critical Appraisal Checklist for Quasi-Experimental Studies (non-randomized experimental studies)

No	Authors	Q1	Q2	Q3	Q4	Q5	Q6	Q7	Q8	Q9
1	Wullimann et al., 2025	+	-	+	+	-	+	+	+	+
